# Supplementary figures and images for: Knowledge attitudes and practices toward seasonal influenza vaccine among pregnant women during the 2018/2019 influenza season in Tunisia
Source: PLoS One. 2022 Mar 22;17(3):e0265390. doi: 10.1371/journal.pone.0265390 (PMC8939791; doi:10.1371/journal.pone.0265390)

S1\_Figure: Sampling method

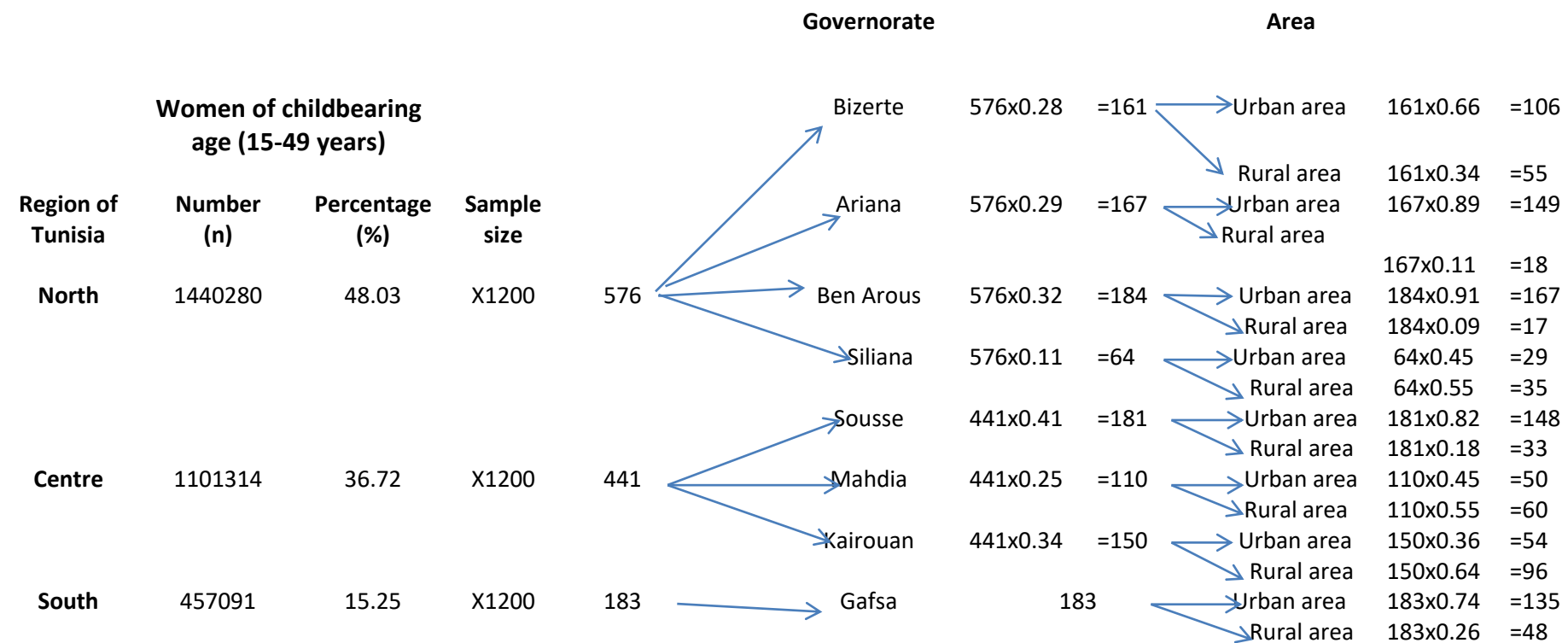

Supplement: S1 Fig — (PDF) [file pone.0265390.s001.pdf]
