# Supplementary material for: Knowledge attitudes and practices toward seasonal influenza vaccine among pregnant women during the 2018/2019 influenza season in Tunisia
Source: PLoS One. 2022 Mar 22;17(3):e0265390. doi: 10.1371/journal.pone.0265390 (PMC8939791; doi:10.1371/journal.pone.0265390)
Supplement: S1 Table — Tunisia, 2018–2019. (PDF) [file pone.0265390.s002.pdf]

S1_Table: Distribution of the selected health care facilities' number by governorate. Tunisia, 2018-2019

Governorate	Primary Healthcare facilities	District Hospital	Regional Hospital	Total
Ariana	3	1	0	4
Ben Arous	12	0	1	13
Bizerte	11	1	1	13
Siliana	6	1	0	7
Sousse	16	2	1	19
Mahdia	6	1	0	7
Kairouan	7	5	1	14
Gafsa	8	0	0	8
Total	69	11	4	84
