# Supplementary material for: Knowledge attitudes and practices toward seasonal influenza vaccine among pregnant women during the 2018/2019 influenza season in Tunisia
Source: PLoS One. 2022 Mar 22;17(3):e0265390. doi: 10.1371/journal.pone.0265390 (PMC8939791; doi:10.1371/journal.pone.0265390)
Supplement: S2 Table — (PDF) [file pone.0265390.s003.pdf]

S2_Table: Distribution of the selected health care facilities' name by governorate, Tunisia, 2018-2019

Governorate	Primary Healthcare facilities	District Hospital	Regional Hospital
**Ariana**	CSB Ettadhamen	HC Ettadhamen	
	CSB Sidi Thabet		
	CSB Soukra		
**Ben Arous**	CSB Ben Arous		HR Ben Arous-Yasminet
	CSB Boumhal		
	CSB Hammam Lif		
	CSB Bir Bey		
	CSB MEHAMEDIA		
	CSB FOUCHANA		
	CSB KHELIDIA		
	CSB Mornag		
	CSB Sidi Mosbeh		
	CSB EL MOUROUJ		
	CSB Rades Foret		
	CSB RADES MELLAHA		
**Bizerte**	CSB 1ER MAY	HC Mateur	HR Menzel Bourguiba
	CSB EL ALIA		
	CSB JOUMIN		
	CSB Mateur		
	CSB Ennajeh		
	CSB Farhat Hached		
	CSB GHZALA-JALTA		
	CSB MENZEL JEMIL		
	CSB CAP ZEBIB		
	CSB BENI ATTA		
	CSB Zarzouna		
**Gafsa**	CSB SIDI HMED ZARROUG		
	CSB DOUALY		
	CSB HAY ENNOUR		
	CSB KEF DERBI MARKEZ		
	CSB KEF DERBI NADHOUR		
	CSB MOUALLA		
	CSB Sidi Boubaker		
	CSB LALLA		
**Kairouan**	CSB El Hajem	HC Bouhajla	
	CSB ZARROUK JEMAA SUD II	HC Chebika	
	CSB Hay Essalem	HC HAJEB EL AYOUN	
	CSB Menchia	HC Haffouz	HR IBN ELJAZZAR
	CSB Ahmed El Garoui	HC Nasrallah	
	CSB Mansoura		

	CSB SBIKHA		
**Mahdia**	CSB Jem	HC EL JEM	
	CSB Tlelsa		
	CSB route Sfax		
	CSB route Sousse		
	CSB Zghabna		
	CSB MCHELET		
**Siliana**	CSB Gaafour	HC Bouaarada	
	CSB SADDINE		
	CSB SNED HADDAD		
	CSB Siliana		
	CSB Hbebsa		
	CSB Bourouis		
**Sousse**	CSB Sidi Salem	HC Bouficha	HR Msaken
	CSB JAWHARA CITE ZAATIR	HC Enfidha	
	CSB Bouhsina		
	CSB Oued Blibene		
	CSB TAIEB MHIRI KALAA KBIRA		
	CSB ENNAGUER		
	CSB Ksibet Soussse		
	CSB MSAKEN NORD		
	CSB Sidi Bouali		
	CSB Ezzouhour		
	CSB Sidi Abdelhamid		
	CSB Riadh		
	CSB ENFIDHA		
	CSB Medina		
	CSB Aouina		
	CSB Mohamed Ali		
