# Supplementary material for: Knowledge attitudes and practices toward seasonal influenza vaccine among pregnant women during the 2018/2019 influenza season in Tunisia
Source: PLoS One. 2022 Mar 22;17(3):e0265390. doi: 10.1371/journal.pone.0265390 (PMC8939791; doi:10.1371/journal.pone.0265390)
Supplement: S3 Table — (PDF) [file pone.0265390.s004.pdf]

S3_Table: Factors associated with willingness to receive influenza vaccine during pregnancy (n=999)[1]

Factors	Willing to receive influenza vaccine n (%)*	Crude OR 95 % CI[2*]	p value
Age group (years)			
18-24 (n=122)	57 (46.6)	1	0.3
25-34 (n=623)	267 (43.7)	0.9 [0.6-1.3]	
≥35 (n=254)	97 (38.8)	0.7 [0.5-1.1]	
Educational level			
Primary school or less (n=226)	100 (44.6)	1	0.8
Secondary school (n=525)	220 (42.7)	0.9 [0.6-1.3]	
Post-secondary or higher (n=242)	100 (42.0)	0.9 [0.6-1.3]	
Employment status			
No (n=634)	277 (44.0)	1	0.4
Yes (n=349)	138 (41.0)	0.9 [0.7-1.2]	
Travel distance from home to antenatal care facility (minutes)			
≥61 (n=24)	12 (47.3)	1	0.9
31-60 (n=162)	68 (44.0)	0.9 [0.3-2.3]	
≤30 (n=801)	337 (42.6)	0.8 [0.3-2.0]	
Term of pregnancy			
First Trimester (n=98)	41 (43.1)	1	0.9
Second Trimester (n=482)	199 (42.0)	0.9 [0.6-1.5]	
Third Trimester (n=412)	176 (43.1)	1.0 [0.6-1.6]	
Number of antenatal care visits completed by time of survey			0.6
>1 (n=901)	378 (42.5)	1	
1 (n=94)	41 (45.2)	1.1 [0.7-1.7]	
Comorbidities prior to this pregnancy			
No (n=813)	321 (39.9)	1	$<10^{-3}$
Yes (n=180)	97 (55.2)	1.9 [1.3-2.6]	
Complications during this pregnancy			
No (n=770)	309 (40.6)	1	0.01
Yes (n=215)	106 (50.7)	1.5 [1.1-2.1]	
Number of pregnancies			
≥3 (n=426)	187 (45.1)	1	0.3
2 (n=269)	118 (43.3)	0.9 [0.7-1.3]	
1 (n=296)	113 (39.0)	0.8 [0.6-1.1]	
History of miscarriage, abortion, or stillbirth			0.5
No (n=731)	305 (42.4)	1	

Yes (n=260)	114 (44.7)	1.1 [0.8-1.5]	
Number of children			
≥3 (n=96)	36 (38.0)	1	
1-2 (n=523)	233 (45.2)	1.3 [0.8-2.2]	0.1
0 (n=321)	121 (38.4)	1.0 [0.6-1.7]	

* weighted according to region, governorate and area of residence

[1] Women who answered ‘I do not Know’ to the question concerning willingness to receive influenza vaccine were excluded (n=154). [2] Confidence interval
