# Supplementary material for: Knowledge attitudes and practices toward seasonal influenza vaccine among pregnant women during the 2018/2019 influenza season in Tunisia
Source: PLoS One. 2022 Mar 22;17(3):e0265390. doi: 10.1371/journal.pone.0265390 (PMC8939791; doi:10.1371/journal.pone.0265390)
Supplement: S1 Appendix — (PDF) [file pone.0265390.s005.pdf]

S1_Appendix: Survey's questionnaire in French language

Questionnaire de l'enquête sur les connaissances, les attitudes et les pratiques des femmes enceintes en Tunisie concernant le vaccin anti grippal, Mars-Mai 2019

Date de l'interview :____________Nom de l'interviewer :
____

Gouvernorat :______________________Délégation : ____________________

Type de la structure sanitaire : Centre de santé de base/ Hôpital de circonscription/ Hôpital régional (encerclez un)

Nom de la structure sanitaire : ____

Milieu : urbain/rural (encerclez un)

I. État de grossesse, antécédents médicaux et consultations prénatales

1. **Quel est le terme de cette grossesse ?_____________Semaines / mois (encerclez un)**
   ☐ Je ne sais pas

2. **Au cours de quel mois de grossesse avez-vous eu votre première visite prénatale ?**
   ________________ Mois

3. **Y compris cette visite, combien de fois avez-vous consulté au cours de cette grossesse ?**
   ____

4. **Y compris cette grossesse, combien de fois avez-vous été enceinte ?**
   ____

5. **Avez-vous déjà fait une fausse couche, une interruption de grossesse ou une mort fœtale ?**
   a. ☐oui
   b. ☐non
   c. ☐Je ne veux pas répondre

6. **Combien d'enfants avez-vous ? __**

7. **AVANT cette grossesse, un médecin, une infirmière ou un autre professionnel de la santé vous a-t-il dit que vous étiez affectée par une des maladies suivantes ?**

a. ☐Diabète
b. ☐Maladie cardiaque
c. ☐Hypertension artérielle
d. ☐Anémie
e. ☐Asthme
f. ☐Allergies
g. ☐ Autres maladies: ______________________
h. ☐je ne sais pas
i. ☐Je ne veux pas répondre
j. ☐Pas de problème de santé

8. PENDANT cette grossesse, un médecin, une infirmière ou un autre professionnel de santé vous a-t-il informé que vous présentiez l'une des affections suivantes ?

a. ☐Diabète
b. ☐Maladie cardiaque
c. ☐Hypertension artérielle
d. ☐Anémie
e. ☐Asthme
f. ☐Allergies
g. ☐Autre : ________________________
h. ☐je ne sais pas
i. ☐Je ne veux pas répondre
j. ☐Pas de problème de santé

9. (Si un problème de santé existe) : Recevez-vous un traitement pour ce problème de santé pendant la grossesse ?

a. ☐oui
b. ☐non
c. ☐Je ne veux pas répondre

10. Avez-vous été hospitalisée pendant cette grossesse ?

a. ☐oui
b. ☐non
c. ☐Je ne veux pas répondre

11. En utilisant votre moyen de transport habituel, combien de minutes vous faut-il pour aller de votre domicile à la structure de santé habituelle pour la consultation prénatale ?

a. ☐Moins de 15 minutes
b. ☐16-30 minutes
c. ☐31-45 minutes
d. ☐46-60 minutes
e. ☐61 à 90 minutes
f. ☐Plus de 90 minutes

g. ☐je ne sais pas
h. ☐Je ne veux pas répondre

II. Histoire générale, connaissances, attitudes vis-à-vis de la grippe et du vaccin de la grippe pendant la grossesse

12. Avez-vous déjà entendu parler de la grippe ?

a. ☐oui
b. ☐non (passez à la question 27)

13. Connaissez-vous quelqu'un qui a présenté une forme grave de la grippe ?

a. ☐Oui
b. ☐Non
c. ☐Je ne me rappelle pas

14. Avez-vous déjà entendu parler du vaccin de la grippe ?

a. ☐oui
b. ☐non (passer à la question 27)

15. Si OUI : Où avez-vous entendu parler du vaccin de la grippe ? (Q.O)

a. ☐membre de la famille, précisez : ____________________
b. ☐ami (e)
c. ☐médecin
d. ☐Pharmacien
e. ☐Sage-femme
f. ☐Autre professionnel de la santé
g. ☐Télévision ou radio
h. ☐Imprimés (journaux. magazines. dépliants)
i. ☐ Internet
j. ☐Autre(s): ______________________________
K.☐ Je ne me rappelle pas

16. Avez-vous déjà été vaccinée au moins une fois contre la grippe ?

a. ☐Oui
b. ☐Non (Passez à la question 19)

17. Avez-vous été vaccinée contre la grippe au cours de cette grossesse ?

a. ☐Oui
b. ☐Non

18. Combien de fois avez-vous reçu le vaccin de la grippe au cours des 5 dernières années ?

a. ☐0 fois
b. ☐une fois

c. ☐2 à 4 fois
d. ☐5 fois
e. ☐Je ne me rappelle pas

19. Quelqu'un vous a-t-il recommandé de recevoir le vaccin de la grippe pendant cette grossesse ?

a. ☐oui
b. ☐Non (passez à la question 21)
c. ☐je ne sais pas (passez à la question 21)

20. Qui vous a recommandé de recevoir le vaccin de la grippe pendant cette grossesse ? (Q.O)

a. ☐Gynécologue
b. ☐Autre médecin
c. ☐Pharmacien
d. ☐Infirmière
e. ☐Sage-femme
f. ☐ assistante sociale
g. ☐Ami(e)
h. ☐Membre de la famille. précisez____________________
i. ☐À la radio. à la télévision
j. ☐Internet
k. ☐Autre(s) : __________________________________
l. ☐ Je ne sais pas

21. Quelqu'un vous a-t-il découragé de recevoir le vaccin de la grippe pendant cette grossesse ?

a. ☐oui
b. ☐Non (passez à la question 23)
c. ☐je ne sais pas (passez à la question 23)

22. Qui vous a découragé de recevoir le vaccin de la grippe pendant cette grossesse ? (Q.O)

a. ☐Gynécologue
b. ☐Autre médecin
c. ☐Pharmacien
d. ☐Infirmière
e. ☐Sage-femme
f. ☐ assistante sociale
g. ☐Ami(e)
h. ☐Membre de la famille, précisez____________________
i. ☐À la radio. à la télévision
j. ☐Internet
k. ☐Autre(s) : __________________________________
l. ☐ Je ne sais pas

23. **Avez-vous entendu dans les médias ou les réseaux sociaux des reportages qui vous feraient hésiter à vous faire vacciner contre la grippe ?**
   a. ☐Oui
   b. ☐Non
   c. ☐Je ne sais pas

24. **Connaissez-vous quelqu'un qui a développé une mauvaise réaction contre le vaccin de la grippe pouvant vous décourager de vous faire vacciner ?**
   a. ☐Oui
   b. ☐Non
   c. ☐Je ne sais pas

25. **Avez-vous suffisamment d'informations sur la sécurité et les effets indésirables des vaccins de la grippe ?**
   a. ☐Oui
   b. ☐Non
   c. ☐Je ne sais pas

26. **Je vais vous citer 8 informations générales sur la grippe et le vaccin de la grippe. Veuillez indiquer votre point de vue en ce qui concerne ces affirmations**

	Tout à fait d' accord	D'accord	Ni en désaccord ni en accord	Pas d'accord	Pas du tout d'accord	Je ne sais pas
a-La grippe est plus dangereuse pour les femmes enceintes que pour les femmes non enceintes						
b-le vaccin de la grippe peut être dangereux pour les femmes enceintes.						
c-le vaccin de la grippe peut être dangereux pour le fœtus						

d-le vaccin de la grippe peut être dangereux pour un nouveau- né.						
e-Le vaccin de la grippe aide à protéger les femmes enceintes contre la grippe.						
f-La vaccination d'une femme enceinte contre la grippe aide à protéger le fœtus.						
g-La vaccination d'une femme enceinte contre la grippe aide à protéger le nouveau-né						
h-Les femmes devraient recevoir le vaccin de la grippe au cours de chaque grossesse						

27. **Voudriez-vous faire vacciner contre la grippe ?**
    a. ☐oui
    b. ☐non
    c. ☐je ne sais pas

28. **Si le vaccin de la grippe saisonnière était recommandé et fourni gratuitement aux femmes enceintes, accepteriez-vous de vous faire vacciner ?**
    a. ☐oui
    b. ☐non
    c. ☐je ne sais pas

29. **Selon vous, quelles sont les 3 principales raisons pour lesquelles vous accepteriez de vous faire vacciner contre la grippe pendant votre grossesse ?**

1-__________________________

2-_________________________________

3- _________________________________

30. **Selon vous, quelles sont les 3 principales raisons pour lesquelles vous refuseriez de vous faire vacciner contre la grippe pendant votre grossesse ?**

1-______________________________

2-______________________________

3- ______________________________

31. **À qui (ou à quelle source) feriez-vous le plus confiance pour vous fournir les informations les plus précises sur les vaccins de la grippe ?**
    a. ☐Médecin
    b. ☐Pharmacien
    c. ☐sage-femme
    d. ☐Infirmier (infirmière)
    e. ☐Assistant(e) social(e)
    f. ☐Amie
    g. ☐membre(s) de famille (s): ____________________
    h. ☐Média
    i. ☐Internet
    j. ☐Autre(s) : _________________________
    k. ☐Je ne sais pas

32. **En général, estimez-vous avoir suffisamment d'informations sur les vaccins, leur sécurité et leurs effets indésirables ?**
    a. ☐Oui
    b. ☐Non
    c. ☐Je ne sais pas

33. **En général, faites-vous confiance aux conseils de votre prestataire de soins de santé (médecin / infirmier(ère)/sagefemme) ?**
    a. ☐ Oui
    b. ☐ Non
    c. ☐ Je ne sais pas

34. **Pensez-vous qu'il existe d'autres moyens de prévention des maladies meilleurs que la vaccination ?**
    a. ☐ Oui
    b. ☐ Non
    c. ☐Je ne sais pas

III. <u>Informations générales et socio-démographiques?</u>

35. Quel âge avez-vous/ date de naissance ? ....

36. Quel est votre état civil ?

a. ☐ célibataire
b. ☐ Mariée
c. ☐ Divorcée
d. ☐ veuve
e. ☐ Autre : ________________________________
f. ☐Je ne souhaite pas répondre

37. Quel est votre niveau d'étude ?

a. ☐ Analphabète
b. ☐ Kotteb
c. ☐ niveau priMayre
d. ☐ niveau secondaire
e. ☐formation professionnelle
f. ☐ niveau universitaire
g. ☐Je ne souhaite pas répondre

38. Est-ce que vous travaillez ?

a. ☐ oui
b. ☐ non
c. ☐ ne souhaite pas répondre

Merci d'avoir répondu à nos questions. Vos réponses nous aideront à comprendre comment répondre au mieux aux besoins des femmes enceintes en Tunisie.
