# Supplementary material for: Knowledge attitudes and practices toward seasonal influenza vaccine among pregnant women during the 2018/2019 influenza season in Tunisia
Source: PLoS One. 2022 Mar 22;17(3):e0265390. doi: 10.1371/journal.pone.0265390 (PMC8939791; doi:10.1371/journal.pone.0265390)
Supplement: S2 Appendix — (PDF) [file pone.0265390.s006.pdf]

S2_Appendix: Survey's questionnaire in English language

Date of interview: _____________**Name of interviewer**: _________________________

Governorate: _____________ **Delegation**__________________

Health Facility's type: Primary healthcare facility/ District Hospital/ Regional Hospital (circle one)

Health Care Facility's Name: _________________________
Urban / rural areas (circle one)

I. Pregnancy status, health history and antenatal care visits

1. **How many weeks/months pregnant are you? _____________ weeks/months (circle one)**
   ☐ I don't know

2. **In which month of pregnancy did you have your first antenatal care visit? _________________ (months)**

3. **Including this visit, how many times have you had an antenatal care visit during this pregnancy? ________________________________**

4. **Including this pregnancy, how many times have you been pregnant?**
5. **Have you ever had a pregnancy that was miscarried, was aborted, or ended in stillbirth?**
   a. ☐ Yes
   b. ☐ No
   c. ☐ I don't want to answer
6. **How many children do you have? _____________________________**
7. **<u>BEFORE</u> this pregnancy. did a doctor, nurse, or other health care worker tell you that you had any of the following conditions? (Check all that apply)**
   a. ☐ Diabetes / "sugar"
   b. ☐ Heart Disease/Condition
   c. ☐ High blood pressure
   d. ☐ Anemia / low iron
   e. ☐ Asthma / wheezing

f. ☐ Allergies
g. ☐ Other condition: ___________________________
h. ☐ I don’t know
i. ☐ I don’t want to answer
j. ☐ No health condition

8. <u>DURING</u> this pregnancy, did a doctor, nurse or other health care worker tell you that you have any of the following conditions? (Check all that apply)

a. ☐ Diabetes / “sugar ”
b. ☐ Heart Disease/Condition
c. ☐ High blood pressure
d. ☐ Anemia / low iron
e. ☐ Asthma / wheezing
f. ☐ Allergies
g. ☐ Other condition: _____________________________
h. ☐ I don’t know
i. ☐ I don’t want to answer
j. ☐ No health condition

9. (If health condition exists): Are you seeking care for this/these health condition(s) during this pregnancy?

a. ☐ Yes
b. ☐ No
c. ☐ I don’t want to answer

10. Have you been hospitalized during this pregnancy?

a. ☐ Yes
b. ☐ No
c. ☐ I don’t want to answer

11. Using your typical method of travel. how many minutes does it take you to get to your antenatal care clinic from your home?

a. ☐ Less than 15 minutes
b. ☐ 16-30 minutes
c. ☐ 31-45 minutes
d. ☐ 46-60 minutes

e. ☐ 61-90 minutes
f. ☐ More than 90 minutes
g. ☐ I don’t know
h. ☐ I don’t want to answer

General history. knowledge. attitudes regarding influenza and influenza vaccine in pregnancy

12. Have you ever heard of seasonal influenza or the flu before?

a. ☐ Yes
b. ☐ No (**skip to the question 27**)

13. Do you know anyone who has been severely ill with influenza?

a. ☐ Yes
b. ☐ No
c. ☐ I don’t remember

14. Have you ever heard of the vaccine against influenza?

a. ☐ Yes
b. ☐ No (**skip to question 27**)

15. If YES: Where did you learn about the influenza vaccine?

a. ☐ Family member. precise ________________________
b. ☐ Friend
c. ☐ Doctor
d. ☐ Pharmacist
e. ☐ Midwife
f. ☐ Other health care worker(s): ________________________
g. ☐ Television or radio
h. ☐ Printed materials (newspapers. magazines. leaflets)
i. ☐ Internet
j. ☐ Other ________________________________
k. ☐ I don’t remember

16. Did you receive the influenza vaccine at least once at the past?

a. ☐ Yes
b. ☐ No (**Skip to question 19**)

17. Did you receive the influenza vaccine during this pregnancy?

a. ☐ Yes
b. ☐ No

18. How many times have you received the influenza vaccine in the last 5 years?

a. ☐ 0
b. ☐ 1
c. ☐ 2-4
d. ☐ 5
e. ☐ I don't remember

19. Has anyone recommended you receive the influenza vaccine during this pregnancy?

a. ☐ Yes
b. ☐ No **(Skip to question 21)**
c. ☐ I don't know **(Skip to question 21)**

20. Who recommended you receive influenza vaccine during this pregnancy?

a. ☐ Doctor (Obs/Gyn)
b. ☐ Other doctor
c. ☐ Pharmacist
d. ☐ Nurse
e. ☐ Midwife
f. ☐ Social worker
g. ☐ Friend
h. ☐ Family member. precise ________________________
i. ☐ Through Radio or TV
j. ☐ Internet
k. ☐ Other________________________
l. ☐ I don't know

21. Has anyone discouraged you from receiving the influenza vaccine during this pregnancy?

a. ☐ Yes
b. ☐ No **(Skip to question 23)**
c. ☐ I don't know **(Skip to question 23)**

22. Who discouraged you from receiving the influenza vaccine during this pregnancy?

a. ☐ Doctor (Obs/Gyn)
b. ☐ Other doctor
c. ☐ Pharmacist
d. ☐ Nurse
e. ☐ Midwife
f. ☐ Social worker
g. ☐ Friend
h. ☐ Family member. precise ________________________
i. ☐ Through Radio or TV
j. ☐ Internet
k. ☐ Other________________________
l. ☐ I don't know

23. Have you heard or read reports in the media or social media that would make you hesitate to be vaccinated with influenza vaccine?

a. ☐ Yes
b. ☐ No
c. ☐ I don't know

24. Do you know anyone who has ever had a bad reaction to influenza vaccine, which may discourage you from getting vaccinated?

a. ☐ Yes
b. ☐ No
c. ☐ I don't know

25. Do you have enough information about the safety and the side effects of influenza vaccines?

a. ☐ Yes
b. ☐ No
c. ☐ I don't know

26. I'm going to quote 8 general information about the influenza and influenza vaccine. please indicate your views regarding these statements

	**Strongly Agree**	**Agree**	**Neither agree nor disagree**	**Disagree**	**Strongly Disagree**	**I don't know**
a-Influenza is more dangerous for pregnant women than no pregnant women	☐	☐	☐	☐	☐	☐
b. Influenza vaccine can be dangerous for pregnant women	☐	☐	☐	☐	☐	☐
c. Influenza vaccine can be dangerous for the fetus	☐	☐	☐	☐	☐	☐
d. Influenza vaccine can be dangerous for the newborn	☐	☐	☐	☐	☐	☐
e. Influenza vaccine helps protect pregnant women against influenza.	☐	☐	☐	☐	☐	☐
f. When a pregnant woman gets the influenza vaccine. it helps protect her unborn baby.	☐	☐	☐	☐	☐	☐
g. When a pregnant woman gets the influenza vaccine. it helps protect her newborn baby.	☐	☐	☐	☐	☐	☐
h. Women should receive influenza vaccine during each pregnancy	☐	☐	☐	☐	☐	☐

27. Would you want to receive the vaccine against influenza?

a. ☐ Yes
b. ☐ No
c. ☐ I don't know

28. If seasonal influenza vaccine was recommended for pregnant women. and provided for free. would you accept to receive the vaccine?

a. ☐ Yes
b. ☐ No
c. ☐ I don't know

29. In your opinion, what are the main 3 reasons you would accept to receive the influenza vaccine during your pregnancy?

1-________________________________

2-________________________________

3- ________________________________

30. In your opinion, what are the main 3 reasons you would refuse to receive influenza vaccine during your pregnancy?

1- ______________________________

2- ______________________________

3- ______________________________

31. Who (or what source) would you trust the most to give you the most accurate information about influenza vaccines?

a. ☐ Doctor
b. ☐ Pharmacist
c. ☐ Midwife
d. ☐ Nurse
e. ☐ Social worker
f. ☐ Friend
g. ☐ Family member(s): ________________________
h. ☐ Media
i. ☐ Internet
j. ☐ Other: ________________________________
k. ☐ I don’t know

32. In general, do you feel you get enough information about vaccines, their safety and their side effects?

a. ☐ Yes
b. ☐ No
c. ☐ I don’t know

33. In general, do you trust the advice of your health care provider (Doctor/Nurse/Midwife)?

a. ☐ Yes
b. ☐ No
c. ☐ I don’t know

34. Do you believe that there are other (better) ways to prevent diseases than through vaccination?

a. ☐ Yes
b. ☐ No

c. ☐ I don’t know

II. General and Demographic Questions

35. What is your age? Date of birth ________________________________

36. What is your marital status?

a. ☐ Single / never married
b. ☐ Married / civil partnership / cohabitating
c. ☐ Divorced / separated
d. ☐ Widowed
e. ☐ Other: _________________________________
f. ☐ I don’t want to answer

37. What is the highest level of education you have completed?

a. ☐ Illiterate
b. ☐ No formal education but can read and write (Kotteb)
c. ☐ Elementary / Primary school
d. ☐ High School / Secondary school
e. ☐ Professional training
f. ☐ University level
g. ☐ I don’t want to answer

38. Are you currently working in a job that results in regular pay or salary?

a. ☐ Yes
b. ☐ No
c. ☐ I don’t want to answer

Thank you for answering our questions. Your answers will help us understand how best to respond to the needs of pregnant women in Tunisia.
