# Supplementary material for: Knowledge attitudes and practices toward seasonal influenza vaccine among pregnant women during the 2018/2019 influenza season in Tunisia
Source: PLoS One. 2022 Mar 22;17(3):e0265390. doi: 10.1371/journal.pone.0265390 (PMC8939791; doi:10.1371/journal.pone.0265390)
Supplement: Attachment — Submitted filename: Response to reviewers-Rebuttal letter PLOS ONE.docx [file pone.0265390.s007.docx]

**Rebuttal letter**

Tunis, August 29, 2021

Plos One Journal

Dear editors and reviewers

I am pleased, on behalf of all co-authors, to submit to your consideration the revised version of our manuscript newly titled “**Knowledge Attitudes and Practices toward seasonal influenza vaccine among pregnant women during the 2018/2019 influenza season in Tunisia**” [Submission ID: [PONE-D-21-08415] - [EMID:8bb08bb7b6d30319] by Sonia Dhaouadi and al for publication in Plos One.

We are grateful to the editor and reviewers for taking time to read the manuscript and for their valuable feedback and comments that we have taken into account.

We respond hereafter point by point to all comments raised. All changes and corrections are highlighted in green in the revised version of the manuscript.

I hope that revisions have improved the quality and the clarity of the paper and that it is now suitable for publication in Plos One.

Sincerely,

Sonia Dhaouadi

National Observatory of New and Emerging Diseases, Tunis, Tunisia

[sonidhaouadi88@gmail.com](mailto:sonidhaouadi88@gmail.com)

**1. Please ensure that your manuscript meets PLOS ONE's style requirements, including those for file naming. The PLOS ONE style templates can be found at https://journals.plos.org/plosone/s/file?id=wjVg/PLOSOne_formatting_sample_main_body.pdf and https://journals.plos.org/plosone/s/file?id=ba62/PLOSOne_formatting_sample_title_authors_affiliations.pdf**

Authors’ response: Done

**2. Thank you for stating in the text of your manuscript "Ethical approval for this survey was obtained from the Biomedical Ethics Committee of the Pasteur Institute of Tunis. Approval was also obtained from the Tunisian Ministry of Health. All women included in the survey were informed about the objectives and modalities of the study. Women who agreed to be interviewed provided written informed consent." Please also add this information to your ethics statement in the online submission form.**

Authors’ response: Done.

**3. Please list the names of all of the public health centers where participants were recruited from.**

Authors’ response: This information was added in Appendix S2.

**4. Please include additional information regarding the survey or questionnaire used in the study and ensure that you have provided sufficient details that others could replicate the analyses. For instance, if you developed the survey or questionnaire as part of this study and it is not under a copyright more restrictive than CC-BY, please include a copy, in both the original language and English, as Supporting Information. If the questionnaire is published, please provide a citation to the (1) questionnaire and/or (2) original publication associated with the questionnaire.**

Authors’ response: Questionnaire survey was added in appendix 3 in both French and English languages (Appendix 3 a-b).

**5. Please include captions for your Supporting Information files at the end of your manuscript, and update any in-text citations to match accordingly. Please see our Supporting Information guidelines for more information: http://journals.plos.org/plosone/s/supporting-information.**

Authors’ response: Done (S1 Table, S1 Appendix, S2 Appendix, S3-a Appendix and S3-b Appendix)

**Please review your reference list to ensure that it is complete and correct. If you have cited papers that have been retracted, please include the rationale for doing so in the manuscript text, or remove these references and replace them with relevant current references. Any changes to the reference list should be mentioned in the rebuttal letter that accompanies your revised manuscript. If you need to cite a retracted article, indicate the article’s retracted status in the References list and also include a citation and full reference for the retraction -notice.**

Authors’ response:

References added:16, 17, 18,20,21, 22,25,26 and 27.

**Reviewer #1: 1. Sampling method - needs more clarity, because the author claims that the sample is a representative of the total population and also able generalize**

Authors’ response: We conducted a multiple stage sampling according to region, governorate and area of residence. More details are show in Methods section (paragraph 3: sampling procedure, line 112-line 126) and in appendix S1.

**2. How they arrive at Eight governorates out of 24 were randomly selected - Please clarify because the representation from all regions was not proportionate**

Authors’ response: Given the limited budget of the survey (limited logistics support and resources), we choose to select the one third of the total 24 Tunisian governorates (24/3=8). Then eight governorates were randomly selected.

**3. This study included only qualitative data that were presented as numbers and percentages - Explain.**

Authors’ response: In order to facilitate the analysis, we decided to categorize them into qualitative variables.

**4. First, the status of influenza vaccination was self-reported by the participants – Self reporting could have been done with EVIDENCE CROSS CHECKED BY VACCINATION CARDS, MCH CARDS ETC. ???**

Authors’ response: There’s not yet an influenza vaccination card in Tunisia (Discussion, line 340-line 341)

**Reviewer #2: 1. The Discussion section paragraph 1 and 2 are the repetitions of Aim and Results of the previous sections. I would consider it as redundant and may i suggest to remove it.**

Authors’ response: We took into account this comment.

**2. Suggested to reason out why the Flu shots uptake are high in U.S,UK and Ireland. This may give a lead to adopt it in Tunisia.**

Authors’ response: This comment was taken into consideration in Discussion part (line 263-line 277)

In those countries (USA and OK), PW are listed and invited to receive the IV. In addition, the healthcare provider can recommend, offer, and administer the vaccine at the same visit as opposed to making a recommendation and referring the patient elsewhere to receive the vaccine. There’s a close collaboration between medical doctors and midwives to ensure the enhance the coverage rate.

**Suggested to correct as Strengths and Limitations and NOT as Strength and Limitations -Plural is required**

Authors’ response: Done

**Reviewer #3: This is an interesting manuscript about pregnant women's attitudes towards having an influenza vaccine in Tunisia. A major concern I have about the paper is that while information about women's attitudes to the vaccine have been sought, it appears that the main reason women do not get the vaccine is because it is not offered to them and it is not free of charge. However, I appreciate that the aim of the manuscript is to form an evidence base for the promotion of the vaccine to pregnant women. I think the manuscript could be enhanced by providing some context in the introduction about what vaccines pregnant women in Tunisia are/are not offered and any associated costs to the women. Even if the vaccine was offered access to getting the influenza vaccine could be a barrier to women, especially those who live rurally. This could also be touched on in the introduction.**

Authors’ response: This idea was taken into account in the introduction. The IV is not free of charge and non-refundable among PW in Tunisia.

**Minor comments**

**Study design - Is March to May winter in Tunisia? Add in details about the season.**

Authors’ response: The 2018/2019 influenza season in Tunisia from 1^st^ October 2018 to end of April 2019. The survey’s period (March-May 2019) coincided with spring season in Tunisia.

**Study population - Is it normal for pregnant women to attend a public health center? Provide a figure on how many women usually attend these clinics.**

Authors’ response: Given the high medical fees of consultation in private sector, PW in Tunisia attended either public and private sector. However, we don’t have exactly the number of PW attending the public health care facilities in public sector.

**Sample size - leave out the equation, just include the sentence about the sample size. This sentence could go with your study population information rather than under its own heading.**

Authors’ response: Done (the sample size paragraph was eliminated).

**Data analysis - Move the sentences together to make two paragraphs.**

Authors’ response: Done

**Ethical considerations - "the" before data is not needed. "will be analyzed" should be "would be analyzed" "is voluntary" should be "was voluntary"**

Authors’ response: Done

**Results**

**First paragraph "Age" should be "age"**

Authors’ response: Done

**Discussion**

**You mention that the uptake of other low-income countries are similar to Tunisia. What are the reasons for this? Is it also because the vaccine is not offered/available? Does it cost money?**

Authors’ response: In Low and Middle income countries, the low IV coverage could be explained by the difficulty of effective implementation of National Strategic Plan for Vaccines among high risk groups, and by the lack of awareness of burden of disease by decisions-makers and by the target population. In Tunisia, it’s more related to the influenza vaccine awareness among pregnant women and Health care workers than the vaccine supplies and health care access. The vaccine is not free of charge and non-refundable among this group.

**Reviewer #4: 1. Subject to qualitative data availability and usage, the authors have used two-year-old data for qualitative study.**

Authors’ response: We conducted a quantitative study.

**2.Sampling among all the three regions are not appropriate, it can be elaborated to give a clear understanding. And what percentage of population has been used in sampling.**

Authors’ response: Please see response to comment 1 reviewer 1.

**3.The study population targeted is all pregnant women more than or equal to 18 years, so less than 18 years of pregnant women cannot be the part of sampling.**

Authors’ response: For ethical reasons (PW aged less than 18 years must to give the written consent of her legal tutor (her parent or her husband) before participation in the survey. So we decided to include only PW aged 18 years and above.

Indeed, we used the published data for the National Institute of Statistics (INS) about the 2014 population census. The repartition of number of women of childbearing age (15-49 years) by age group was as follows (five-year interval): 15-19, 20-24,25-29,30-34,35-39,40-44 and 45-49 years. We can’t use the age >=18 for the sampling.

**4.Elaborate the criteria on which the status of influenza vaccination was reported by the targeted population.**

Authors’ response: We took into consideration this comment in method section (line 134-136)

**Reviewer #5: This paper presents the results of a national survey of attitudes toward influenza vaccination among pregnant women attending antenatal care in Tunisia. The authors report that the majority of women had heard of the vaccine, but only a small minority had been vaccinated, which was related to concerns about the safety and effectiveness of the vaccine. The authors conclude that health care professionals working in antenatal care can increase vaccination rates by encouraging their patients to get vaccinated.**

**The paper is well-written, with a well-justified aim and an interesting discussion. My main concern is that it is difficult to evaluate whether the results are generalizable to the study population (i.e., to all pregnant women attending antenatal care in the Tunisian public health care sector), as the sampling procedure is not described in detail and the response rate has not been reported. My specific comments can be found below.**

**Abstract/Introduction:**

1. **Should vaccine uptake be included in the aims?**

Authors’ response**:** Yes, assess influenza vaccine uptake was added in the aims au suggested. (Abstract, Background part, line 38).

**2. In reference 16, I cannot find the WHO’s target of 80% of pregnant women being vaccinated.**

Authors’ response**:** Reference 16 was updated as mentioned.

**Methods:**

3. **The authors write that sampling was stratified by region, but how was the number of governorates sampled from each region determined?**

Authors’ response**:** We applied the percentage of Women of childbearing age in each region (North, Center and South) to determine the number of selected governorates in each region:

48.03% (North)*8=4 governorates.

36.72% (Center)*8=3 governorates

15.25% (South)*8=1 governorate

The governorates were then randomly selected in each region.

More details are shown in appendix 1.

4. **What was the second sampling stage? Were hospitals/health care centers sampled within governorates? According to the text, after governorates had been sampled, “stratified sampling was conducted based on the selected governorates”. Could the authors please clarify what was sampled and what the stratification variable(s) was (were)?**

Authors’ response**:** We conducted a multiple stage sampling according to 3 stratified variables: region, governorate and area of residence. More details are show in Methods section (paragraph 3: sampling procedure, line 112-line 126) and in appendix S1**.**

5. **How were hospitals/health care centers within governorates sampled?**

Authors’ response: All public health care centers (primary and secondary health care centers) with antenatal care service were included (Methods, line 124-126)

6. **Were all women attending antenatal care at the sampled hospitals/health care centers asked to participate, or was a sample of women taken?**

Authors’ response: Yes, all women attending antenatal care at the sampled hospitals/health care centers at the time of the survey were asked to participate.

7. **The authors write that the sample was self-weighted and that “the sample size was distributed according to the distribution of the target population in the different regions.” Does this mean that the number of sampled patients in each governorate was proportional to the size of the total population size?**

Authors’ response: Yes; that the number of sampled patients in each governorate was proportional to the size of the women of childbearing age’s population size.

.

8. **Was the sampling design taken into account in the data analysis? If not, please explain why.**

Authors’ response: No, we did a self-weighted sampling taking into account the distribution of the woman of childbearing age according to region governorates and area of residence as we don't have such information for pregnant women.

9. **The authors explain that the interviewers were trained. However, it is unclear who the interviewers were. Were they, for example, nurses or other health care professionals already working at the hospitals/health care centers, or were they external staff?**

Authors’ response: The interviewers were health care worker in public health sector (Methods, line 130-131).

10. **In the Data Analysis section, I think “qualitative data” should say “qualitative variable”, as the data are quantitative (i.e., numbers), but the variables are qualitative (i.e., ordinal/nominal).**

Authors’ response: Done

**11. In the Data Analysis section, it would be good to mention that odds ratios and 95% confidence intervals were calculated.**

Authors’ response**:** This comment was taken into consideration in Methods part (line 154-155).

**Results:**

**12. How many health care centers/hospitals were sampled?**

Authors’ response**: 84**

**13. How many health care centers/hospitals were asked to participate in the survey? How many were willing to participate?**

Authors’ response**:** The sampled 84 health care centers/hospitals were asked to participate in the survey and were willing to participate.

**14. How many women were assessed for eligibility? How many were considered eligible? How many were excluded? What was the response rate in the survey?**

Authors’ response**:** The number of pregnant women who were approached to participate in the survey as well as the response rate were added in the first paragraph of result part (line 169-line 172).

**15. In the second paragraph, should the word “nearly” be replaced with “more than”?**

Authors’ response**:** Nearly three quarter (74.7%) was replaced by more than half as suggested (line 173 in Results section).

**16. In the third paragraph, please include not only the percentage, but also the number, of women who received the vaccine in the past or during their current pregnancy.**

Authors’ response**:** The number of women who received the vaccine at least once in the past and during the current pregnancy was added (line 183-184).

**17. In the third paragraph, should “FIV” be “FV”?**

Authors’ response**:** Flu vaccination (FV) was replaced by influenza vaccination (IV) in all the manuscript.

**18. In the sixth paragraph, should the word “except” be deleted or replaced with another word?**

Authors’ response**:** except was replaced by both of women (Results, line 192).

**19. In the last paragraph of the Results section, the authors mention two reasons why women refused getting vaccinated (fear of side effects and concerns regarding self-harm/harm to the fetus). Was this question open-ended? If it was, did the authors consider categorizing all responses, instead of just two? If it was not open-ended, what were the possible responses? The answers “fear of side effects” and “concerns regarding self-harm/harm to the fetus” seem identical to me. Could these answers be combined?**

Authors’ response**:**

**-**Question related to the main reasons of IV refuse was an open-ended question (question 30 in the questionnaire). There were three main reasons of IV refuse:

* Fear of side effects” and “concerns regarding self-harm/harm to the fetus (72,1%)

* Concerns about vaccine efficacy (16.2%)

* Natural immunity inducted by the infection is better than the immunity inducted by the vaccine (11.2%) (Results section line 214-217).

**20. In the last paragraph of the Results section, it says that the main reason that patients were not willing get vaccinated was concerns about adverse effects. However, in Table 2, it looks like vaccinated and non-vaccinated women differ more in their beliefs about vaccine effectiveness than vaccine safety. Could this result mean that concerns about lack of effectiveness are an even more important reason for the women’s unwillingness to get vaccinated than concerns about adverse effects?**

Authors’ response**:** The question related to the main reasons to refuse IV was an open ended question (question 31 in the questionnaire). Questions related to the vaccine efficacy/vaccine safety presented in Table 3 was a multiple choice question (question 26 in the questionnaire, based on five points Likert scale). The response of interviewed women differed regarding the priority according to the type of question. It seems that vaccine safety concerns were more important than vaccine efficacy concerns when it was a spontaneous response (open ended question), unlike to multiple choice question (inducted response) where vaccine efficacy concerns were more priority.

**Discussion/Conclusion:**

**21. Would it be interesting to mention that only 48% of women claim that they would take the vaccine if it were recommended by a health care professional and provided free of charge, which is substantially lower than the target of 80% vaccinated among pregnant women, even if this would be a large improvement from the current 4%? In addition, would it be interesting to mention that as many as 75% say they would be willing to take it if it was recommended by a doctor, but only 34% if it was recommended by another health care professional?**

Authors’ response**:** This idea was taken into account in discussion section (line 284-290).

**22. Page 8, Paragraph 3: Is “avail” the right word here?**

Authors’ response**:** Avail was replaced by uptake as suggested (Discussion line 311).

**23. Page 8, last paragraph: Most questions do not seem to be open-ended, as they are multiple-choice.**

Authors’ response**:** Yes, it was a Likert scale question based on 5-point agreement scale (Table 3).

**Table 2:**

**24. Is there a round-off error in the odds ratio for the first question (“Do you know anyone who has been severely ill with influenza?”)? I get an odds ratio of 0.95 (=136*360/272*189), which rounds up to 1.0.**

Authors’ response**:**

OR was rounded to 1.0 in Table 2 as suggested.

**References**

**25. Parts of the references are in French.**

Authors’ response**:**

The English title was added in those references (Ref 9 and 40).

**Thank you again for taking the time to share your insightful feedback.**
