# Supplementary material for: Knowledge attitudes and practices toward seasonal influenza vaccine among pregnant women during the 2018/2019 influenza season in Tunisia
Source: PLoS One. 2022 Mar 22;17(3):e0265390. doi: 10.1371/journal.pone.0265390 (PMC8939791; doi:10.1371/journal.pone.0265390)
Supplement: Attachment — Submitted filename: Response to reviewers-Rebuttal letter second revision.docx [file pone.0265390.s008.docx]

**Rebuttal letter**

Tunis, November 26, 2021

Plos One Journal

Dear editors and reviewers

I am pleased, on behalf of all co-authors, to submit to your consideration the2^nd^ revised version of our manuscript newly titled “**Knowledge Attitudes and Practices toward seasonal influenza vaccine among pregnant women during the 2018/2019 influenza season in Tunisia**” [Submission ID: [PONE-D-21-08415] - [EMID:8bb08bb7b6d30319] by Sonia Dhaouadi et al for publication in Plos One.

We are grateful to the editor and reviewers for taking time to read the manuscript and for their valuable feedback and comments that we have taken into account.

We respond hereafter point by point to all comments raised. All changes and corrections are highlighted in green in the revised version of the manuscript.

I hope that revisions have improved the quality and the clarity of the paper and that it is now suitable for publication in Plos One.

Sincerely,

Sonia Dhaouadi

National Observatory of New and Emerging Diseases, Tunis, Tunisia

[sonidhaouadi88@gmail.com](mailto:sonidhaouadi88@gmail.com)

00216 95334250

**Major issues:**

**1. Methods, Line 113: How did the authors determine that 8 governorates should be sampled? In their rebuttal, they explain that this was determined by budget constraints, as 8 corresponds to 1/3 of Tunisia’s 24 governorates. Pease add this information to the manuscript.**

Authors’ response: We took into consideration your suggestion and we added this information to the manuscript (Methods, lines 116-117).

**2. Methods, Lines 112-113: It could be clarified how the number of governorates sampled per region was determined. I know this information is provided in Lines 120-123, but the authors provide a much clearer explanation in their rebuttal (i.e. 8 * % of Tunisia’s women of childbearing age living in a region). I recommend that the authors be as clear in the manuscript as they are in their rebuttal.**

Authors’ response: Your suggestion was taken into account. Please see Methods section, lines 117-123.

**3. Methods, 118-119: It is unclear how area of residence (urban/rural) was relevant to the sampling procedure. According to the authors’ rebuttal, all public health centers in the sampled governorates were included. If so, shouldn’t area of residence be irrelevant?**

Authors’ response: We apologize; it seems that we made an error when explaining the sampling method in the rebuttal letter. We meant that all public health centers were targeted by the selection procedure. Indeed, as we explained in the response to comment 4, not all public health centers were selected. In addition, we took into consideration area of residence in the self-weighting process in order to enhance similarity of distribution between our study population and the target population. It should be noted that area of residence refers to the residence of PW and not to the location of the health care facility. Some PW living in rural area may visit health care centers in urban area and vise-versa.

**4. Methods, Sampling procedure: In the authors’ rebuttal, it sounds like they performed region-stratified, cluster sampling, meaning that they first randomly sampled governorates from each region and then included all pregnant women attending any public health center in the sampled governorates. If so, this could be clarified in the Sampling Procedure section.**

Authors’ response: We reiterate our apology for the error when explaining the sampling method in the rebuttal letter. In fact, we performed a stratification according to region. Governorates were randomly selected (four in the North, three in the Center and one in the South). However, we did not proceed to a cluster sampling. Indeed, in each governorate we randomly selected health care facilities among those offering antenatal care visits as the survey progressed. Every day, we randomly selected one center among all those that offer medical care consultations for PW on that day. The selected center will not be selected again.

On the day of their visit, investigator(s) were asked to randomly select participants from the exhaustive list of PW consulting the selected health care facility on that day. The number of PW approached to participate to the survey in each health care facility depended on the availability of human and logistic resources.

It should be noted that a pilot study was performed in order to assess the comprehensiveness of questionnaire items, to identify ambiguous questions and to estimate the maximum number of questionnaires that could be properly administrate by one investigator which was equal to 20. The sampling procedure was clarified as suggested in sampling procedure (Please see lines 114-136).

**5. Results, Line 169: The number of survey respondents was 1200, which is identical to the calculated sample size reported in the Methods section. This seems unlikely if the authors really did recruit all pregnant women attending any public health center during a 3-month period. Are the authors’ sure they didn't recruit consecutive patients and then stop when the calculated sample had been reached?**

Authors’ response: Thank you for your comment. In fact, you are right we stopped recruitment of participants when the needed sample size was achieved.

**6. S1 Appendix: The sampling method illustrated here looks like it is stratified sampling by region, governorate, and area of residence. As mentioned above, however, only region seems to have been a stratification variable.**

Authors’ response: We totally agree with you; region was the only stratification variable.

Details regarding sampling procedure were clarified on Methods section.

**7. Rebuttal: The authors explain that they did not take the sampling procedure into account during the analysis due to a lack of information to do so. However, ignoring cluster sampling in an analysis can lead to confidence intervals that are too narrow. Have the authors considered this possibility?**

Authors’ response: We totally agree with you. Not considering cluster sampling in analysis can lead to narrow confidence intervals. However, in the present study we did not proceed to a cluster sampling. In fact, we randomly selected daily health care facilities among those offering antenatal care visits as the survey progressed. On the day of their visit, investigator(s) were asked to randomly select participants from the exhaustive list of PW consulting the selected health care facility on that day. The number of PW approached to participate to the survey in each health care facility depended on the availability of human and logistic resources. The details were shown in sampling procedure (Methods). Please see lines (114-136).

**Minor issues:**

**8. Abstract, Line 48: The lower bound of the confidence interval is 57.7% here, but it is 57.6% in the main text (Results, Line 182). Please check this.**

Authors’ response: Thank you for your comment. The lower bound of the confidence interval is in fact 57.6%. We corrected the mistake in the abstract (Please see line 48).

**9. Abstract, Lines 48-49: The text states that 4.4% of women who were aware of the vaccine received it during their current pregnancy; however, this percentage seems to be calculated based on all 1157 included women (51/1157=4.4%). The same was done in Results, Lines 182-184, both for women who had received the vaccine during the current pregnancy and for those who had received it sometime in the past. Please check this.**

Authors’ response: Your suggestion was taken into consideration (Please see line 49 in Abstract and line 199 in Manuscript).

**10. Results, Line 163: Could the ID numbers be linked to personal identifiers? If so, the data would be coded/pseudo-anonymized rather than anonymized.**

Authors’ response: Data were coded anonymized and the ID number was not linked to personal identifiers.

**11. Results, Lines 181-182: Is there a round-off error in the percentage of women who had heard of influenza, 85.5%? I get 988/1157=84.4%. Is there an error in the calculation of women who had heard of the vaccine, 60.3%? I get 694/1157=60.0%.**

Authors’ response: In calculation, we only took into account valid percentage.

Women who had heard of influenza: n=988; missing data=2;

The valid percentage=988/**1155**=85.5%; 95% CI [83.4%–87.5%].

Women who had heard of influenza vaccine:

n=694; missing data=7; The valid percentage=694/1150=60.3%; 95% CI [57.5%–63.2%].

**12. Results, Line 187: Consider adding the number of women who would be willing to receive the vaccine, instead of just reporting the percentage.**

Authors’ response: This suggestion was taken into consideration: Results, line 207. (Yes=421, No=578, I don’t know=154, missing data=4; the valid percentage=421/1153=36.5%)

**13. Discussion, Line 307: How was 6.5% calculated (numerator/denominator)? According to Table 2, 72 patients reported having enough information about side effects. Is 72 the numerator used?**

Authors’ response: The calculated percentage regarding information about side effects of IV: 6.5% (valid percentage) =75/1146 (Yes=75; No=1015; I don’t know=56; missing data=11). This information was added in Results section: lines 196-197.

Concerning table 2, we took into account only PW who answered Yes or No about willing to receive IV in the next pregnancy, participants who answered I don’t know about willingness to receive IV were excluded (Please see footnote Table 2). Participants who answered I don’t Know about having enough information about side effects were also excluded. Among the 75 PW who reported they had enough information about IV side effects, two answered I don’t know when asked about willingness to receive IV in the next pregnancy, so they were not included in Table 2.

**14. References: There are still French words here.**

Authors’ response: All references were corrected into English language.

**Response to Editor Dr. Deshayne**:

We reported 95% confidence interval of crude odds ratio in text (Results section) to be more informative as suggested (lines 211, 212, 216, 218 and 219)

**Thank you again for taking the time to share your insightful feedback and for taken into consideration our request that our manuscript be considered for inclusion in your ongoing Influenza collection (https://collections.plos.org/collection/influenza/)**
