# Supplementary material for: Knowledge attitudes and practices toward seasonal influenza vaccine among pregnant women during the 2018/2019 influenza season in Tunisia
Source: PLoS One. 2022 Mar 22;17(3):e0265390. doi: 10.1371/journal.pone.0265390 (PMC8939791; doi:10.1371/journal.pone.0265390)
Supplement: Attachment — Submitted filename: Response to reviewers-Rebuttal letter PLOS ONE Feb 2022.docx [file pone.0265390.s009.docx]

**Rebuttal letter**

Tunis, February 14, 2021

Plos One Journal

Dear editors and reviewers,

I am pleased, on behalf of all co-authors, to submit to your consideration the revised version of our manuscript newly titled “**Knowledge Attitudes and Practices toward seasonal influenza vaccine among pregnant women during the 2018/2019 influenza season in Tunisia**” [Submission ID: [PONE-D-21-08415] - [EMID:8bb08bb7b6d30319] by Sonia Dhaouadi and al for publication in Plos One.

We are grateful to the editor and reviewers for taking time to read the manuscript and for their valuable feedback and comments that we have taken into account.

We respond hereafter point by point to all comments raised. All changes and corrections are highlighted in green in the revised version of the manuscript.

I hope that revisions have improved the quality and the clarity of the paper and that it is now suitable for publication in Plos One.

Sincerely,

Dr Sonia Dhaouadi

National Observatory of New and Emerging Diseases, Tunis, Tunisia

[sonidhaouadi88@gmail.com](mailto:sonidhaouadi88@gmail.com)

**Minor comments:**

**1. Page 4: In their rebuttal letter, the authors confirm my previous suspicion that the only stratification variable was region. However, now that the authors have further clarified their work, I think that the sampling procedure was also stratified by area of residence, as healthcare centers were selected from both urban and rural areas.**

Authors’ response**:** Thank you for your comment, area of residence was also considered as suggested as variable of stratification (Methods, sampling procedure, line 122).

**2. Page 4, Lines 126-127: The authors explain that patients were randomly selected at healthcare centers, but the authors may also want to explain how this was done in practice. For example, did the study staff enter the total number of patients on the patient list into a computer, which then returned a certain number of randomly selected patients, such as patients 2, 5, and 6 on the list?**

Authors’ response: This comment was taken into account in Methods section, sampling procedure, lines 129-130

**3. Page 5: The authors have not addressed my question of whether it is appropriate to ignore the sampling procedure in the analysis. If I am not mistaken, Appendix S1 contains all the information required to incorporate this information. (The percentages at each stage of the sampling procedure could be used to construct sampling weights, could they not?)**

Authors’ response: Thank you for your comment, this proposition was taken into consideration in the results section, all percentages, crude ORs and their 95% confidence interval were weighted as mentioned according to region, governorate and area of residence.

**Thank you again for taking the time to share your insightful feedback.**
